# Robotic transanal minimally invasive surgery (R-TAMIS): current evidence in the treatment of early rectal neoplasia

**DOI:** 10.1007/s00384-024-04645-4

**Published:** 2024-05-09

**Authors:** Niall J. O’Sullivan, Hugo C. Temperley, John Larkin, Jacob J. McCormick, Emanuele Rausa, Paul McCormick, Alexander Heriot, Brian J. Mehigan, Satish Warrier, Michael E. Kelly

**Affiliations:** 1 Department of Radiology, St. James’s Hospital, Dublin, Ireland; 2 School of Medicine, Trinity College Dublin, Dublin, Ireland; 3 The National Centre for Advanced Medical Imaging (CAMI), St. James’s Hospital, Dublin, Ireland; 4 Department of Surgery, St. James’s Hospital, Dublin, Ireland; 5 Department of Surgery, Peter MacCallum Cancer Centre, Melbourne, 3000 Australia; 6 Unit of Hereditary Digestive Tract Tumours, Fondazione IRCCS Istituto Nazionale Dei Tumori, Milan, Italy; 7 Trinity St. James Cancer Institute, Dublin, Ireland

**Keywords:** Robotic surgery, Minimally invasive surgery, Rectal neoplasia, TAMIS

## Abstract

**Introduction:**

Robotic transanal minimally invasive surgery (R-TAMIS) was introduced in 2012 for the excision of benign rectal polyps and low grade rectal cancer. Ergonomic improvements over traditional laparoscopic TAMIS (L-TAMIS) include increased dexterity within a small operative field, with possibility of better surgical precision. We aim to collate the existing data surrounding the use of R-TAMIS to treat rectal neoplasms from cohort studies and larger case series, providing a foundation for future, large-scale, comparative studies.

**Methods:**

Medline, EMBASE and Web of Science were searched as part of our review. Randomised controlled trials (RCTs), cohort studies or large case series (≥ 5 patients) investigating the use of R-TAMIS to resect rectal neoplasia (benign or malignant) were eligible for inclusion in our analysis. Quality assessment of included studies was performed via the Newcastle Ottawa Scale (NOS) risk of bias tool. Outcomes extracted included basic participant characteristics, operative details and histopathological/oncological outcomes.

**Results:**

Eighteen studies on 317 participants were included in our analysis. The quality of studies was generally satisfactory. Overall complication rate from R-TAMIS was 9.7%. Clear margins (R0) were reported in 96.2% of patients. Local recurrence (benign or malignant) occurred in 2.2% of patients during the specified follow-up periods.

**Conclusion:**

Our review highlights the current evidence for R-TAMIS in the local excision of rectal lesions. While R-TAMIS appears to have complication, margin negativity and recurrence rates superior to those of published L-TAMIS series, comparative studies are needed.

**Supplementary Information:**

The online version contains supplementary material available at 10.1007/s00384-024-04645-4.

## Introduction

Robotic transanal minimally invasive surgery (R-TAMIS) was introduced in 2012 for the excision of benign rectal polyps and low grade rectal cancer [1]. Robotic platforms offer the benefits of wristed articulation, 3D optics and improved ergonomics, combatting issues posed by traditional laparoscopic TAMIS (L-TAMIS) [2, 3]. Theoretical benefits of R-TAMIS include superior oncologic resections and increased dexterity, via fine motion scaling and articulated instruments respectively [4]. Similarly, the use of a robotic platform averts the need of an active surgical assistant in the already confined work space [5].

Despite promising reports in the literature, there is a void of large comparative studies, particularly comparing peri-operative and oncological outcomes between R-TAMIS and L-TAMIS [5]. In addition, most of the current literature on R-TAMIS are small series or individual case reports, limiting the power and interpretation of results [6–13]. We aim to collate the existing data surrounding the use of R-TAMIS to treat rectal neoplasms from cohort studies and larger case series, providing a foundation for future, large-scale, comparative studies.

## Methods

### Study design and reporting guidelines

This study is a systematic review of randomised and non-randomised trials and follows the Preferred Reporting Items for Systematic Reviews and Meta-Analyses (PRISMA) reporting guidelines.

### Search strategy

The following databases were searched as part of the systematic review in October 2023: Medline, EMBASE and Web of Science. The terms included (R-TAMIS OR TAMIS OR Transanal OR Trans-anal) AND (Rect*). The last date of search was 1st of October 2023. The grey literature was also searched to further identify other suitable publications.

### Inclusion criteria

The studies were assessed for eligibility based on the following inclusion criteria. Randomised controlled trials (RCTs), cohort studies or large case series (≥ 5 patients) investigating the use of R-TAMIS to resect rectal neoplasia (benign or malignant) were eligible for inclusion in our analysis.

### Study selection, data extraction and critical appraisal

A database was created using the reference managing software EndNote X9™. Two researchers (NOS and HCT) reviewed outputs from the searches independently of each other.

Initially, duplicates were removed. The study titles were then screened and assessed for potential relevance. The abstracts of selected potential studies were then read and assessed for eligibility for inclusion, based on the inclusion/exclusion criteria detailed above. The rejected studies were grouped together in the database by their reason for exclusion. The full texts of the abstracts deemed eligible for inclusion were then further analysed using the same criteria.

In order to extract and store data efficiently, the Cochrane Collaboration screening and data extraction tool, Covidence, was used [14]. Data was collected by two reviewers (NOS and HCT) independently, using the following headings: study details, study design, population, intervention, comparison groups and outcomes. Conflicts between the two reviewers were resolved following an open discussion and a final decision by the senior author (MK).

Assessment of potential biases for the non-RCT studies was assessed using the Newcastle–Ottawa scale (NOS) risk of bias tool, and the results were tabulated. This assessment tool grades each study as being ‘satisfactory’ or ‘unsatisfactory’ across various categories. We assigned stars to evaluate study quality: 7 stars, ‘very good’; 5–6 stars, ‘good’; 3–4 stars, ‘satisfactory’; and 0–2 stars, ‘unsatisfactory’. The critical appraisal was completed by two reviewers independently (NOS and HCT), where once again a third reviewer (MK) was asked to arbitrate in cases of discrepancies in opinion.

### Systematic review registration

Our systematic review was registered on PROSPERO in October 2023 (470657).

## Results

### Search results

The literature search described above yielded a total of 782 results (Supplementary Material S1). Following the removal of 163 duplicates, 619 studies were screened. After the initial screening, 53 abstracts were reviewed and assessed for eligibility, of which 30 were selected for full-text review. From these 30 full texts, a total of 18 studies met the inclusion criteria and were included in our analysis. 

### Methodological characteristics and quality of studies

Seven of the included studies were cohort studies; one prospective, six retrospective [1, 5, 15–19]. The remaining 11 studies were case series [11, 20–29]. Lee et al. provided a comparison of outcomes between L-TAMIS and R-TAMIS in their comparative study [5]. Table 1 summarises the methodological characteristics of the included studies.

**Table 1:** Methodological characteristics of the included studies

**Study**	**Country**	**Journal**	**Impact factor**	**Study type**
Atallah et al. [20]	USA	Techniques in Coloproctology	3.5	Case series
Baker et al. [15]	Australia	Colorectal Disease	3.4	Prospective cohort study
Fok et al. [21]	Australia	International Journal of Medical Robotics and Computer Assisted Surgery	2.5	Case series
Hompes et al. [22]	UK	British Journal of Surgery	5.6	Case series
Huang et al. [16]	Taiwan	Asian Journal of Surgery	3.5	Retrospective cohort study
Lee et al. [5]	USA	Surgical Endoscopy	3.5	Retrospective cohort study
Liu et al. [18]	USA	Surgical Endoscopy	3.5	Retrospective cohort study
Liu et al. [23]	USA	Techniques in Coloproctology	3.5	Case series
Liu et al. [17]	USA	Updates in Surgery	2.7	Retrospective cohort study
Lo et al. [24]	USA	Surgical Endoscopy	2.5	Case series
Marks et al. [1]	USA	Diseases of Colon and Rectum	3.9	Retrospective cohort study
Ngu et al. [25]	Taiwan	International Journal of Medical Robotics and Computer Assisted Surgery	2.5	Case series
Paull et al. [26]	USA	Journal of Robotic Surgery	2.3	Case series
Ruiz et al. [29]	Spain	Cirugia Espanola	1.9	Case series
Tomassi et al. [19]	USA	Diseases of the Colon and Rectum	3.9	Retrospective cohort study
Warren et al. [11]	Australia	Techniques in Coloproctology	3.5	Case series
Wassef et al. [27]	USA	Journal of Gastrointestinal Surgery	3.2	Case series
Yao et al. [28]	China	Surgical Innovation	1.5	Case series

In regards to quality assessment, seven studies were ‘very good’, nine studies were ‘good’, two studies were ‘satisfactory’ and zero studies were ‘unsatisfactory’ when scored using the Newcastle–Ottawa Scale risk of bias tool. Supplementary Material S2 summarises the results of our risk of bias assessment.

### Participant characteristics

The total number of participants from the 18 included studies was 317. The median follow-up across studies was 30 months (range 12–63). Basic participant characteristics are presented in Table 2. Indications for R-TAMIS included resection of benign neoplasia, early malignant lesions, lesions unresectable by endoscopy and select T2/T3 lesions in patients unfit for or unwilling to proceed with radical surgery.

**Table 2:** Basic participant characteristics

**Study**	**Patients**	**Male:Female**	**Age (mean +/− SD**)	**BMI**	**Pre-operative diagnosis**
Atallah et al. [20]	9	4:5	65	26	Benign neoplasia of rectum not amenable to endoscopic retrieval, selected T1 lesions, T2/T3 lesions in unfit patients
Baker et al. [15]	11	6:5	70 +/− 12	26	‘Rectal lesions identified on colonoscopy’ 6 Invasive, 5 other
Fok et al. [21]	5	5:0	67	-	Benign rectal polyps or T1 tumours
Hompes et al. [22]	15	8:8	65 +/− 14	28 +/− 5	‘Rectal lesions’
Huang et al. [16]	23	11:12	60 +/− 13	24 +/− 3	Early rectal cancer T1 or cCR after CRTx
Lee et al. [5]	40 (L-TAMIS 21, R-TAMIS 19)	-	L-TAMIS: 65 R-TAMIS: 67	L-TAMIS: 29 R-TAMIS: 30	Benign and malignant rectal lesions
Liu et al. [18]	34	23:11	63 +/− 10	29 +/− 6	Early-stage rectal neoplasm (uTis or uT1N0M0), T1 carcinoid, incomplete endoscopic resection of polyps, partial resection for palliative control of rectal bleeding in metastatic disease
Liu et al. [23]	5	3:2	58	-	Endoscopically unresectable polyps or T1 rectal cancers
Liu et al. [17]	28	20:8	62 +/− 10	30 +/− 6	Endoscopically unresectable polyps or low-risk malignancies (no LVI, no TB, well-moderate differentiation, favourable location)
Lo et al. [24]	16	-	63	-	Endoscopically unresectable tumours
Marks et al. [1]	26	13:13	62	27	Benign polyp, T1 cancer, selected T2 cancer after neoadjuvant therapy
Ngu et al. [25]	6	2:4	62 +/− 8	27	Patients with cCR after neoadjuvant therapy for rectal cancer
Paull et al. [26]	21	13:8	Si: 56 +/− 13 FCD: 55 +/− 17	Si: 27 +/− 5 FCD: 28 +/− 7	T0-T1 N0
Ruiz et al. [29]	9	5:4	72 +/− 8	-	Benign rectal lesions or T1 with good prognostic criteria
Tomassi et al. [19]	58	-	-	28 +/− 5	uT1N0, uT2N0, Stage 3 with cCR, polyps, carcinoids, GIST
Warren et al. [11]	8	-	-	-	-
Wassef et al. [27]	19	11:8	68 +/− 10	31	Incomplete endoscopic resection, not amenable neither to endoscopic resection nor conventional transanal excision
Yao et al. [28]	24	10:14	59 +/− 12	24	12 AC (7cCR, 4cT1, 1cT2), 3 CIS, 2 inconclusive, 3 NE, 3 TVA, 1 endometriosis

*cCR* clinical complete response, *CRTx* chemoradiotherapy, *LVI* lymphovascular invasion, *GIST* gastrointestinal stromal tumour, *AC* adenocarcinoma, *CIS* carcinoma in situ, *NE* neuroendocrine, *TVA* tubulovillous adenoma

### Operative details

DaVinci robotic platforms (S, Si, SP or Xi) were used in all but two of the included studies reporting platform models [1, 5, 11, 15–20, 22–25, 27–29]. Fok et al. utilised the Medrobotics Flex Robotic System on their cohort, whereas Paull et al. compared outcomes between the DaVinci Si and Medrobotics Flex Robotic System in their respective studies on R-TAMIS [21, 26]. Patient positioning throughout studies included lithotomy, modified lithotomy, prone jack-knife and hockey-stick decubitus. Variable positions within individual studies were attributed to surgeon preference or tumour location (anterior, posterior, lateral). Fifteen studies reported using the Applied Medical GelPOINT Path Transanal Access Platform. Hompes et al. use a transanal glove port, generally considered a safe, inexpensive and readily available alternative tool in transanal surgery [22]. Operative details are demonstrated in Table 3. The overall mean operative time across the included studies was calculated as 110 +/− 22 min.

**Table 3:** Operative detail**s**

**Study**	**Robotic system**	**Position**	**TAMIS port**	**Operative time (mean +/− SD)**	**Estimated blood loss**
Atallah et al. [20]	DaVinci S/Si	Modified lithotomy	GelPOINT	124	35 ml
Baker et al. [15]	DaVinci Xi	Lithotomy	GelPOINT	67 +/− 17	-
Fok et al. [21]	Medrobotics Flex System	Lithotomy	-	142	-
Hompes et al. [22]	DaVinci Si	-	Glove port	109 +/− 40	-
Huang et al. [16]	DaVinci Si/Xi	Prone jack-knife	GelPOINT	112 +/− 59	-
Lee et al. [5]	DaVinci Si/Xi	Prone jack-knife	GelPOINT	L-TAMIS: 100 R-TAMIS: 102	L-TAMIS: 15 ml R-TAMIS: 5 ml
Liu et al. [18]	DaVinci Xi	Prone jack-knife or lithotomy	GelPOINT	100	-
Liu et al. [23]	-	Lithotomy	GelPOINT	-	-
Liu et al. [17]	DaVinci Xi	Lithotomy	GelPOINT	133 +/− 47	-
Lo et al. [24]	DaVinci Xi	Prone jack-knife or lithotomy	GelPOINT	87	17.5 ml
Marks et al. [1]	DaVinci SP	Modified lithotomy	GelPOINT	199 +/− 116	10 +/− 46 ml
Ngu et al. [25]	DaVinci Xi	Modified lithotomy	GelPOINT	125 +/− 43	Minimal
Paull et al. [26]	DaVinci Si or Medrobotics Flex System	Prone jack-knife or high lithotomy	GelPOINT	Si: 167 +/− 84 FCD: 110 +/− 40	Si: 37.5 +/− 38.3 FCD: 9.5 +/− 13.6
Ruiz et al. [29]	DaVinci Si	Lithotomy	GelPOINT	76 +/− 23	37.4 +/− 8.6 ml
Tomassi et al. [19]	DaVinci Xi/Si	Hockey-stick decubitus OR prone jack-knife OR lithotomy	GelPOINT	82 +/− 41	-
Warren et al. [11]	DaVinci Xi	Prone jack-knife	GelPOINT	-	-
Wassef et al. [27]	-	Prone jack-knife	-	97 +/− 17	Minimal
Yao et al. [28]	DaVinci Xi/Si	Lithotomy or prone jack-knife	GelPOINT	140 +/− 45	Minimal

### Peri-operative outcomes

Peri-operative outcomes are illustrated in Table 4. Complications were divided into major (Clavien Dindo ≥ 3) and minor (Clavien Dindo < 3). Overall complication rate was 9.7% (*n* = 29/300). Minor and major complications were observed in 7% (*n* = 21/300) and 2.7% (*n* = 8/300), respectively. Of the major complications, four were bleeding requiring endoscopic intervention, one pelvic abscess requiring drainage, one poor wound healing requiring re-operative, one peritoneal violation requiring conversion and repair and one post-operative stenosis requiring dilatation.

**Table 4:** Peri-operative details

**Study**	**Length of stay (days)**	**Minor complications**	**Major complications**	**Readmission**	**Follow-up**
Atallah et al. [20]	1.4 +/− 1.1	-	-	-	14 +/− 9 months
Baker et al. [15]	1.8 +/− 0.9	0	1 (bleeding requiring endoscopic clipping)	0	2 weeks
Fok et al. [21]	0.8 +/− 0.6	0	0	0	30 days
Hompes et al. [22]	1.7 +/− 1.2	1	0	0	-
Huang et al. [16]	4.3 +/− 2.6	0	0	0	9.6 months
Lee et al. [5]	L-TAMIS: 10 h R-TAMIS: 10.5 h	L-TAMIS: 1 R-TAMIS: 1	0	0	-
Liu et al. [18]	1	1 (C. Diff infection requiring antibiotics)	0	0	188 +/− 209 days
Liu et al. [23]	0	0	0	0	30 days
Liu et al. [17]	82% 0 days	1 (post-operative hypotension)	1 (POD9 bleeding requiring endoscopy)	1	24 +/− 9 months
Lo et al. [24]	2.8 +/− 2.8	2 (stool incontinence, abscess requiring antibiotics)	1 (poor wound healing requiring re-operation)	1	80 days
Marks et al. [1]	73% 0 days	3 (wound dehiscence requiring antibiotics)	1 (pelvic abscess requiring IR drain)	0	4 +/− 4 months
Ngu et al. [25]	4.2 +/− 1.3	0	0	0	21 +/− 9 months
Paull et at. [26]	-	-	2 (peritoneal violation requiring conversion and repair, rectal stenosis requiring dilatations)	0	-
Ruiz et al. [29]	2.5 +/− 1.2	2 (urinary retention, respiratory infection)	0	0	19 +/− 2 months
Tomassi et al. [19]	90% 0 days	4 (2 urinary retention, nausea, suture line dehiscence requiring antibiotics)	2 (bleeding requiring endoscopy)	-	-
Warren et al. [11]	-	-	-	-	-
Wassef et al. [27]	79% 0 days	6 (4 diarrhoea, pain, urgency)	0	-	-
Yao et al. [28]	4.6 +/− 2	0	0	0	24 +/− 10 months

### Histopathological and oncological data

Tumour specifications were similar across the included studies. Specimen fragmentation occurred in only four cases. The pooled margin positivity rate across studies was 3.8% (*n* = 11/286). Out of the reported, the overall local recurrence (benign or malignant) occurred in 2.2% (*n* = 5/232) of patients within the specified follow-up period. The recurrence locations included the proctectomy scar site, anorectal junction and surgical bed. The histopathological and oncological data is demonstrated in Table 5.

**Table 5:** Histopathological and oncological data

**Study**	**Lesion diameter (cm)**	**Distance to anal verge (cm)**	**Surface area (cm**^**2**^**)**	**Fragmentation**	**Positive margins**	**Recurrence**
Atallah et al. [20]	3.1 +/− 1.4	6.8 +/− 2.3	-	0	1/9	0
Baker et al. [15]	3.8 +/− 1.2	7.3	-	1/11	1/11	-
Fok et al. [21]	3.2	8.3	-	0	1/5	-
Hompes et al. [22]	-	7.3 +/− 2	8 +/− 5.9	-	2/15	-
Huang et al. [16]	2.5	5 +/− 1.5	-	0	2/23	0
Lee et al. [5]	-	L-TAMIS: 7.8 R-TAMIS: 8.2	L-TAMIS: 17 R-TAMIS: 17	-	L-TAMIS: 2/23 R-TAMIS: 1/19	-
Liu et al. [18]	2.6 +/− 1.1	8.6 +/− 3.6	7.7 +/− 9.8	0	0	-
Liu et al. [23]	3	9.7	8.9	0	0	-
Liu et al. [17]	4.1 +/− 3.1	7.8 +/− 3.9	-	-	1/28	0
Lo et al. [24]	4.1	-	-	0	0	-
Marks et al. [1]	2.8 +/− 1.3	3.5 +/− 7.5	-	0	0	0
Ngu et al. [25]	2.8 +/− 1.2	6.5 +/− 1.5	-	0	0	0
Paull et al. [26]	-	Si: 11.1 +/− 3.8 FCD: 9.6 +/− 3.6	-	-	0	0
Ruiz et al. [29]	-	6.4 +/− 1.5	21.5 +/− 12.1	0	0	0
Tomassi et al. [19]	3.3 +/− 1.7	8.8 +/− 2.5	-	1/58	3/58	3/58 (adjacent to proctotomy scar, surgical bed, recurrent polyp)
Warren et al. [11]	-	-	-	0	-	0
Wassef et al. [27]	2.8 +/− 0.9	7.5 +/− 3.5	-	2/19	0	1/19: site of excision
Yao et al. [28]	2.8 +/− 1.1	6.8 +/− 2.1	-	0	0	1/24: anorectal junction

### Cost

Only two of the studies included in our analysis reported costs associated with R-TAMIS [5, 22]. Lee et al. reported direct costs of $3562 and $4440.92 of L-TAMIS and R-TAMIS respectively in their comparative study [5]. Hompes et al. reported a far more economical cost of €837 for a single R-TAMIS case in their institute [22]. Neither study incorporated the capital costs of robotic platforms in their calculations.

## Discussion

Transanal minimally invasive local excision remains an acceptable treatment option for early-stage rectal tumours (T1, N0) within 3–8 cm of the anal verge in the absence of nodal involvement on local staging [30]. While this procedure has traditionally been performed laparoscopically, recent evidence has suggested R-TAMIS as a safe alternative, with better articulation and superior visualisation [24]. We sought to synthesise currently available data on R-TAMIS, to assess its safety and efficacy profile on cases to date.

Our review was comprised of 317 patients from 18 cohort studies or large case series (≥ 5 patients) highlighting practical aspects of R-TAMIS in addition to peri-operative and oncological outcomes. Overall complication rate from R-TAMIS was 9.7%. Clear margins (R0) were reported in 96.2% of patients. Local recurrence occurred in 2.2% of patients during the specified follow-up periods. A recent systematic review of laparoscopic TAMIS outcomes in over 1200 cases by Kim et al. found an overall complication rate of 18.4% (*n* = 222/1205) [31]. Margin positivity rates were also elevated at 8.6% (*n* = 101/1173). Finally, local recurrence was reported as 7.2% (*n* = 54/746). While these findings suggest a potential benefit of R-TAMIS, these studies were observational and non-comparative, making side-by-side comparison difficult and unreliable.

To date, only one study has been published directly comparing outcomes between the laparoscopic and robotic-assisted approach in patients undergoing transanal local excision of rectal lesions. In their single-centre review of 40 consecutive patients, Lee et al. compared the two approaches in terms of peri-operative outcomes, histopathological outcomes and cost [5]. Their review found no significant difference between L-TAMIS and R-TAMIS in any outcome other than cost, which was significantly higher in the R-TAMIS group (median direct cost $3562 vs. $4440.92). A breakdown of cost was not provided in this study.

Our study has several limitations. Despite only including cohort studies or large case series, the majority of included studies contained small numbers of participants, which are by default highly susceptible to potential selection bias. Similarly, a lack of comparative studies made meta-analysis of results and a statistical comparison between surgical approaches not feasible. Further studies on larger populations, ideally in the form of randomised control trials or matched cohorts, are required before conclusions can be drawn on the superiority of one modality over another. Nevertheless, R-TAMIS appears to be a safe and feasible alternative to L-TAMIS, in the transanal local excision of rectal tumours.

## Conclusion

Our review highlights the current evidence for R-TAMIS in the local excision of rectal lesions. While R-TAMIS appears to have complication, margin negativity and recurrence rates superior to those of L-TAMIS published elsewhere in the literature, accurate conclusions cannot be drawn in the absence of comparative studies. Future research focusing on comparing these two approaches will shed light on best practices.

## Supplementary Information

Below is the link to the electronic supplementary material.

Supplementary file1 (DOCX 43.8 kb)

## Data Availability

No datasets were generated or analysed during the current study.
