# Supplementary material for: Robotic transanal minimally invasive surgery (R-TAMIS): current evidence in the treatment of early rectal neoplasia
Source: Int J Colorectal Dis. 2024 May 9;39(1):71. doi: 10.1007/s00384-024-04645-4 (PMC11082025; doi:10.1007/s00384-024-04645-4)
Supplement: Supplementary file 1 — Supplementary file1 (DOCX 43.8 kb) [file 384_2024_4645_MOESM1_ESM.docx]

**Supplementary Material**

**Supplementary material S1: Study selection.** A PRISMA Flowchart of the selection of relevant publications included in this review

**Records identified through database search**
(n = 782)

**Records excluded**
(n = 589)

Deleted based on title = 566

Deleted based on abstract = 23

**Studies included in qualitative synthesis**
(n = 18)

**Full-text articles assessed for eligibility**
(n = 30)

**Records after duplicates removed**
(n = 619)

**Records screened**
(n = 619)

Identification

Screening

**Full-text articles excluded with reasons**
(n = 12)

**Study Design = 6**

*Case reports (7)*

*Small Case series (5)*

Eligibility

Included

| Author | **Selection** | | | | **Comparability.** | **Outcome** | | | **Quality** |
| --- | --- | --- | --- | --- | --- | --- | --- | --- | --- |
|  | **Representativeness of the exposed cohort** | **Sample size (<20 = no star)** | **Open cases only included** | **Ascertainment of the exposure** | **The subjects in different outcome groups are comparable** | **Assessment of outcome** | **Less than 10% missing data?** | **Average Follow up period (> 12 months)** |  |
| Atallah 2015 | **** | / | **** | **** | / | **** | **** | / | 5 |
| Baker 2019 | **** | / | **** | / | **** | **** | **** | **** | 6 |
| Fok 2022 | **** | /** ** | **** | **** | **** | **** | / | / | 5 |
| Hompes 2014 | **** | / | / | **** | **** | / | **** | / | 4 |
| Huang 2020 | **** | **** | **** | **** | **** | **** | **** | **** | 8 |
| Lee 2018 | **** | ** ** | **** | **** | **** | **** | / | **** | 7 |
| Lee 2019 | **** | **** | **** | **** | **** | **** | **** | **** | 8 |
| Liu 2018 | **** | **** | **** | **** | **** | **** | **** | **** | 8 |
| Liu 2020 | **** | / | / | / | **** | / | **** | **** | 4 |
| Liu 2022 | **** | **** | **** | **** | **** | **** | **** | / | 7 |
| Lo 2021 | **** | /** ** | **** | **** | / | **** | **** | **** | 6 |
| Marks 2021 | **** | ** ** | **** | **** | **** | / | **** | **** | 7 |
| Ngu 2018 | **** | / | **** | **** | **** | **** | **** | **** | 7 |
| Paull 2019 | **** | **** | **** | / | / | **** | / | **** | 5 |
| Ruiz 2017 | **** | / | / | **** | **** | **** | **** | / | 5 |
| Tomassi 2018 | **** | **** | **** | / | **** | **** | **** | **** | 7 |
| Warren 2018 | **** | / | **** | **** | **** | **** | / | **** | 6 |
| Wassef 2022 | **** | / | **** | **** | / | **** | **** | **** | 6 |
| Yao 2020 | **** | **** | **** | **** | **** | / | **** | / | 6 |

**Supplementary material S2: Risk of bias assessment (Newcastle-Ottawa scale)**
